# Dual Repressive Function by Cip1, a Budding Yeast Analog of p21, in Cell-Cycle START Regulation

**DOI:** 10.3389/fmicb.2020.01623

**Published:** 2020-07-09

**Authors:** Pan Li, Xueqin Liu, Zhimin Hao, Yanrong Jia, Xiangdong Zhao, Debao Xie, Jingao Dong, Fanli Zeng

**Affiliations:** ^1^College of Life Sciences, Hebei Agricultural University, Baoding, China; ^2^State Key Laboratory of North China Crop Improvement and Regulation, Baoding, China

**Keywords:** budding yeast, cell cycle, G1/S transition, Cip1, Ccr4, Caf120, p21

## Abstract

Cip1, a newly identified yeast analog of p21, is a Cln3-CDK inhibitor that negatively regulates cell-cycle START. However, its function remains poorly understood. In this study, we found that deletion of *CLN3* did not result in bypass of G1-phase arrest caused by Cip1 overexpression. Cip1 depletion in *cln3*-null mutants significantly advanced the timing of Cln2 expression, supporting the idea that Cip1 represses START in a Cln3-independent manner. We set to search for novel Cip1 interacting proteins and found that Ccr4, a known START regulator, and its associated factor Caf120, interact with Cip1. Ccr4-Caf120 acts redundantly with Cdk1-Cln3 to inhibit Whi5-mediated regulation of START. This interaction was conserved between human Ccr4 and p21. In addition, deletion of *WHI5* robustly suppressed G1-phase arrest caused by Cip1 overexpression. We conclude that Cip1 negatively regulates START by acting as a dual repressor of Ccr4 in parallel with Cln3.

## Introduction

In budding yeast, as well as in mammals, cell proliferation is primarily regulated at the G1/S-phase transition in response to a variety of environmental and internal signals (Bertoli et al., 2013). Regulation at this stage, which determines whether and when a cell will enter the division cycle, converges on a major conserved surveillance mechanism known as “START” in yeast and the “Restriction Point” in mammals (Johnson and Skotheim, 2013). START has evolved to be a critical decision-making point in late G1 phase that ensures a “no-return” cell-cycle entry. Loss of strict control of cell-cycle initiation results in uncontrolled proliferation and developmental diseases (Massague, 2004; Nojima, 2004).

START is promoted through activation of the cell size-dependent regulator Cln3-Cdk1, which relocalizes to the nucleus upon activation and phosphorylates the retinoblastoma (Rb) ortholog Whi5 (Wittenberg and Reed, 2005; Palumbo et al., 2016). Phosphorylated Whi5 is excluded from the nucleus, allowing activation of SBF. In turn, SBF is responsible for expression of late G1 genes, including cyclins encoded by *CLN1* and *CLN2*, resulting in cell-cycle entry. This mechanism is analogous to the regulation of E2F transcription by the tumor suppressor Rb, which constitutes a key barrier to carcinogenesis in human cells (Bruin et al., 2004; Costanzo et al., 2004; Cooper, 2006).

In mammals, the tumor suppressor p21, a downstream target of p53, is the main factor responsible for negatively regulating the Cln3-Cdk1 ortholog CycD/E-CDK and suppressing unwanted cell division at the G1/S transition (Georgakilas et al., 2017). This strategy for regulating CDK activity is conserved from yeast to human, but previously no p21 or p53 homolog had been identified in yeast. Recently, the *Saccharomyces cerevisiae* protein Cip1 was shown to regulate G1/S transition in both the unperturbed and stressed cell cycle (Chang et al., 2017; Zhang Z. et al., 2017); accordingly, it was proposed as a functional analog of p21. Cip1 was originally identified as a G1-Cdk1 inhibitor that negatively regulates G1/S transition (Ren et al., 2016). Cip1 associates with G1-Cdk1s, including Cln3-Cdk1, and inhibits their activities both *in vitro* and *in vivo* (Ren et al., 2016; Chang et al., 2017). Targeting of Cln3-Cdk1 by Cip1 prevents Whi5 phosphorylation and thereby prevents the G1/S transition (Chang et al., 2017).

Cells have evolved strict and redundant pathways for crucial aspects of cell-cycle control. We sought to determine whether Cip1 regulates the G1/S transition in another manner, distinct from its previously identified regulation of Cln3-Cdk1. Cip1 overexpression blocks cell-cycle entry, arrests cells at G1 phase, and significantly increases cell size; all of these events are Cln3-dependent (Chang et al., 2017).

In this study, we found that *CLN3* deletion could not suppress cell-cycle arrest caused by Cip1 overexpression, indicating that another target of Cip1, in parallel with Cln3-Cdk1, is involved in START control. Proteomic screening identified Ccr4-Caf120 complex as a novel downstream target of Cip1. The conserved Ccr4-Not complex, involved in mRNA biogenesis, fine-tunes the START program by modulating *CLN1* and *CLN2* expression through destabilization of *WHI5* mRNAs (Manukyan et al., 2008). Consistent with this, the Ccr4-Not complex is essential for the G1/S transition in stressed cells (Westmoreland et al., 2004). However, the regulatory function of Ccr4-Not in cell-cycle control remains largely unknown. Our results demonstrate that Cip1 blocks the cell cycle at START by acting as a dual repressor of Ccr4-Caf120 and Cdk1-Cln3.

## Results and Discussion

### Cip1 Overexpression Causes Significant G1 Arrest in *cln3* Mutants

**FIGURE 1:**
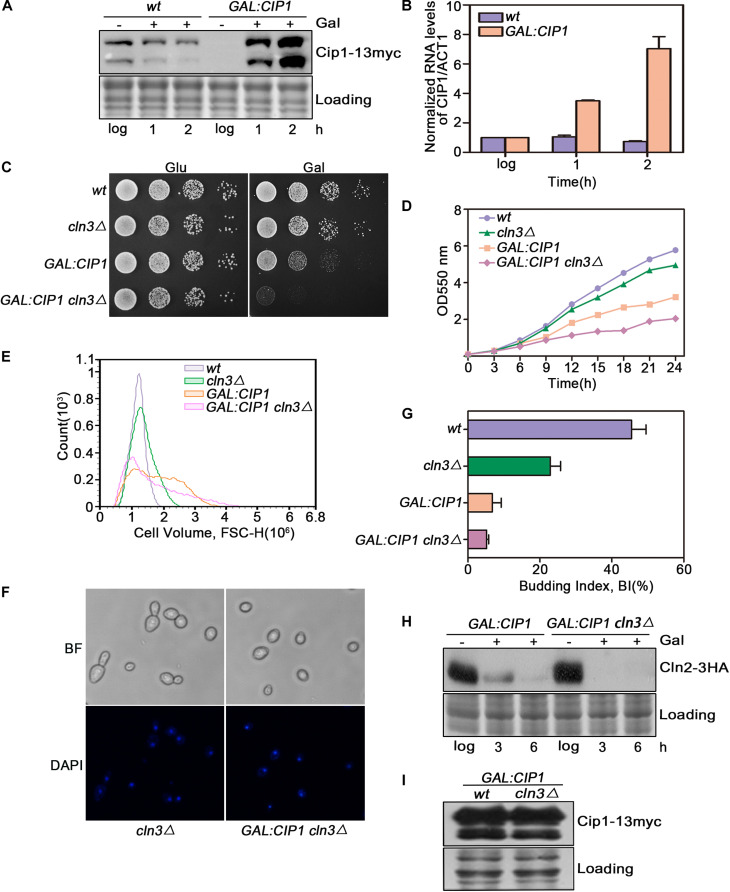
Cln3 depletion does not bypass G1 phase arrest caused by Cip1 overexpression. **(A)** Western blot analysis of Cip1 protein level in wild-type (YFL100) and galactose-induced Cip1 protein level in *GAL:CIP1* (YFL97) cells. **(B)** Real-time quantitative PCR analysis of relative amount of *CIP1* mRNA in wild-type (YFL100) and *GAL:CIP1* (YFL97) cells after the addition of galactose. Mean values were from three independent replicates. Value was arbitrarily set at 1 for log samples. **(C)** 10-fold serial dilutions from cultures of wild-type (YFL3), *cln3*Δ (YXQ7), *GAL:CIP1* (YFL97), and *GAL:CIP1 cln3*Δ (YXQ11) were spotted onto YPD and YPG, and incubated at 30°C for 3 days. **(D)** The same indicated strains were grown in YPR. Galactose (2%) was added to induce overexpression of Cip1, and growth curves were generated. **(E)** Cell volume distributions for the indicated strains are shown (20,000 cells analyzed/sample). Galactose (2%) was added to induce overexpression of Cip1, and samples were collected after 3h induction. **(F,G)** Cell-cycle distribution of cells after 3 h since the addition of galactose. Pictures show bright field images and DAPI staining of nuclei. Budded cells were counted and expressed as a percentage of total cells. **(H)** The levels of Cln2 protein were analyzed by western blotting at 3 and 6 h after addition of galactose. **(I)** Western blot analysis of Cip1 protein level in cells grown in YPG.

Cip1 is an inhibitor of Cln3-Cdk1. To determine whether it might also play a Cln3-independent repressive role, we overexpressed Cip1 and monitored the effect on G1/S control. *CIP1* was overexpressed under the GAL1 promoter. Upon galactose addition, Cip1 was functional largely induced as monitored at protein and mRNA levels shown in Figures 1A,B. In a wild-type *CLN3* background, overexpression of Cip1 significantly inhibited cell growth, consistent with our previous observations (Ren et al., 2016). However in *cln3*-null cells, high levels of Cip1 resulted in a stronger growth defect (Figures 1C,D).

We found that this growth defect was due to a stronger inhibition of cell-cycle START in *cln3*-null mutants. In yeast overexpressing Cip1, more than 90% of cells arrested before START as large unbudded cells with a single nucleus, despite *CLN3* deletion (Figures 1E–G). Expression of *CLN2*, a G1/S marker gene, was completely blocked in cells overexpressing Cip1 (Figure 1H). The overexpression extends of Cip1 in both wild-type and *cln3*-null cells is identical as controlled in Figure 1I. Therefore, START regulation by Cip1 might involve mechanisms independent of Cln3.

### Cip1 Negatively Regulates START in a Cln3-Independent Manner

**FIGURE 2:**
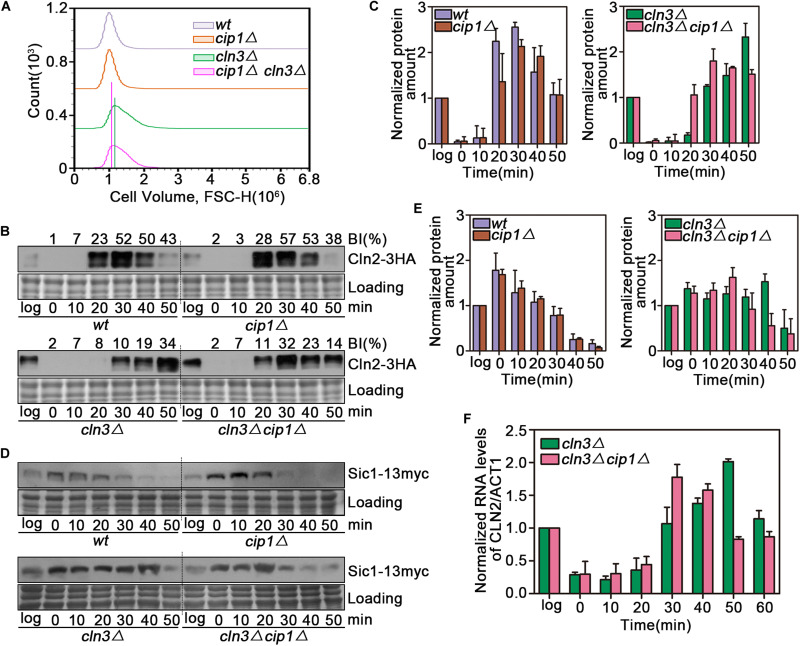
Effect of Cip1 depletion on START timing in a *cln3* mutant strain. **(A)** Flow cytometry analysis of the cell volume distributions for the indicated strains (20,000 cells analyzed/sample). **(B)** Cultures of Cln2-3HA (YZZ6), *cip1*Δ Cln2-3HA (YZZ20), *cln3*Δ Cln2-3HA (YXQ8), and *cln3*Δ *cip1*Δ Cln2-3HA (YXQ9) were grown to mid-log phase in YPD and synchronized in pre-START G1. The cells were released from the α-factor arrest such that they entered the cell cycle synchronously. Samples were taken at the indicated time points (min). TCA whole-cell extracts were analyzed by immunoblotting with anti-HA (Cln2) antibodies. A Coomassie brilliant blue-stained region of the same membrane used for immunoblotting is shown as a loading control. The budding index (BI) monitoring the cell cycle progression were shown on the top of time course panels. **(C)** Graphs show quantification of Cln2, normalized against the loading control. Mean values were from three independent replicates. Value was arbitrarily set at 1 for log samples. **(D)** Cultures of Sic1-13myc (YLP139), *cip1*Δ Sic1-13myc (YLP149), *cln3*Δ Sic1-13myc (YLP155), and *cln3*Δ *cip1*Δ Sic1-13myc (YLP138) were grown to mid-log phase in YPD and synchronized in pre-START G1. The cells were released from the α-factor arrest such that they entered the cell cycle synchronously. Samples were taken at the indicated time points (min). TCA whole-cell extracts were analyzed by immunoblotting with anti-myc (Sic1) antibodies. A Coomassie brilliant blue-stained region of the same membrane used for immunoblotting is shown as a loading control. **(E)** Graphs show quantification of Sic1, normalized against the loading control. Mean values were from three independent replicates. Value was arbitrarily set at 1 for log samples. **(F)** Normalized relative amount of *CLN2* mRNA vs. *ACT1* control mRNA in *cln3*Δ (YXQ8) and *cln3*Δ *cip1*Δ cells (YXQ9). Mean values were from three independent replicates. Value was arbitrarily set at 1 for log samples.

We observed a significant cell size decrease in *cln3*Δ *cip1*Δ double mutants compared to larger *cln3*Δ cells. We quantified this cell size alteration of each cell population by FACS analysis. As shown in Figure 2A, *cln3*Δ *cip1*Δ double mutants displayed a clear shift in the overall distribution toward small cell volumes compared to *cln3*Δ single mutants. We next asked whether deletion of *CIP1* and *CLN3* would advance START timing relative to single deletion of *CLN3*. For this purpose, we used Cln2 accumulation as the marker of START entry. Cln2 rapidly accumulated 20 min after wild-type cells were released from α-factor-induced pre-START arrest (Figures 2B,C). A similar expression pattern was observed in *cip1*Δ mutants. In *cln3*-mutant cells, which are defective in START activation, Cln2 expression was delayed for at least 10 min relative to wild-type cells. However, the delay in Cln2 expression and cell budding were rescued by double deletion of *CLN3* and *CIP1* (Figures 2B,C).

To confirm this result, we monitored the stability of Sic1, whose disappearance is a marker of START execution. Sic1 began to be degraded at 20 min and disappeared 40 min after release from α-factor arrest in both wild-type and *cip1*Δ cells (Figures 2D,E). In *cln3* mutants, Sic1 was stable for the duration of the experiment. By contrast, in double-mutant (*cln3*Δ *cip1*Δ) cells, START entry was advanced by more than 10 min.

To confirm that these observations were due to an effect on the START transcriptional program, we compared *CLN2* mRNA levels between *cln3*Δ and *cln3*Δ *cip1*Δ mutants. G1/S gene expression, as reflected by *CLN2* mRNA levels, was advanced by ∼20 min in *cln3*Δ *cip1*Δ double mutants relative to *cln3*Δ cells (Figure 2F). These observations strongly indicate that Cip1 has a Cln3-independent repressive function in START control.

### Ccr4 Interacts With Cip1/p21

**FIGURE 3:**
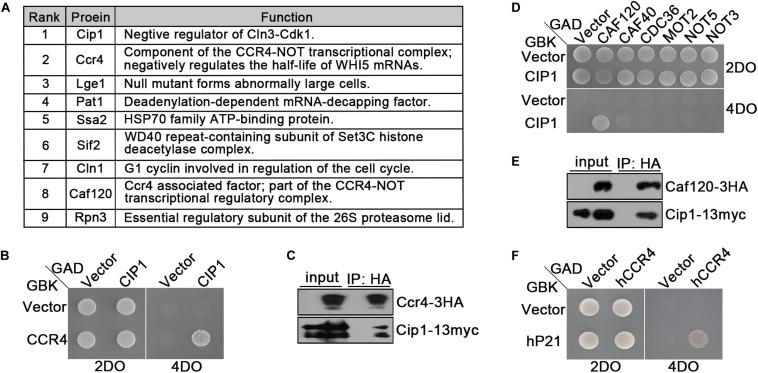
Ccr4 interacts with Cip1/p21. **(A)** Co-IP identified a range of Cip1 interacting proteins. Exponentially growing cells YFL100 (Cip1-13myc) and untagged control cells (YFL3) were harvested and extracted, and the extracts were immunoprecipitated using anti-myc antibodies. The precipitated proteins were separated by SDS-PAGE, and the differential bands were analyzed by mass spectrometry. In addition to Cip1 itself, the eight most abundant interacting proteins are shown in the table. The rank value is based on the frequency of peptide occurrence. **(B)** Cip1 interacted with Ccr4 in the yeast two-hybrid assay. Yeast cells transformed with the indicated plasmids were grown at 30°C on the control 2DO (SD-leucine and -tryptophan) and high-stringency 4DO (SD-adenine, -histidine, -leucine, and -tryptophan) plates. **(C)** Exponentially growing cultures of Cip1-13myc control cells (YFL100) and Cip1-13myc Ccr4-3HA cells (YLP41) were harvested. Ccr4-3HA was immunopurified from the indicated extracts using anti-HA antibodies. Copurified Cip1 was analyzed with anti-myc antibodies. **(D)** Yeast two-hybrid assay revealed that Cip1 robustly interacts with Caf120 but not with other Not subunits. **(E)** Co-IP assay showing that Cip1 interacts with Caf120. The procedure was similar to that described for Panel C. **(F)** Yeast two-hybrid assay showing that p21 interacts with human Ccr4.

As shown above, *CLN3* deletion could not suppress cell-cycle arrest induced by Cip1 overexpression, and *cln3*Δ *cip1*Δ double mutants exhibited accelerated entry into START. Hence, we performed proteomic assays to identify novel proteins associated with Cip1. The top nine hits are listed in Figure 3A. Most of the proteins in this list, such as Ccr4, Lge1 and Cln1 (Partridge et al., 1997; Westmoreland et al., 2003; Manukyan et al., 2008; Zhang W. et al., 2017), were previously implicated in G1/S cell-cycle regulation, confirming the specificity of our co-immunoprecipitation and mass spectrometry assays for Cip1-interacting proteins. Cln1 is a paralog of the G1/S cyclin Cln2, which binds to Cip1. Ccr4 is an essential component of the Ccr4-Not transcriptional complex, which negatively regulates the half-life of *WHI5* mRNA and influences the timing of START (Manukyan et al., 2008).

Interestingly, our proteomic assays identified two subunits of the Ccr4-Not complex, Ccr4 and Caf120, as hits (Figure 3A). We verified the interaction between Cip1 and Ccr4 by using east two-hybrid assays (Figure 3B). We also carried out an experiment complementary to the one that led to the identification of Ccr4 as a Cip1-associated protein; we immunoprecipitated Ccr4-3HA from exponentially growing cells and probed for Cip1 among the associated proteins. Cip1 was reproducibly associated with Ccr4 (Figure 3C). Subsequent yeast two-hybrid assays and co-IP also confirmed the interaction between Cip1 and Caf120 (Figures 3D,E). Moreover, this interaction was specific to Caf120 and did not extent to other members of the Not complex, including Caf40, Cdc36, Mot2, Not5, and Not3 (Figure 3D).

Because Cip1 is functionally similar to human p21 (Ren et al., 2016; Chang et al., 2017), we speculated that the Ccr4 interaction might be conserved between the two proteins. Indeed, in a yeast two-hybrid assay, we identified a robust interaction between p21 and hCcr4 (Figure 3F). This result indicates that Ccr4 in both yeast and human cells is a bona fide target of Cip1/p21.

### Ccr4 Is a Novel Target of Cip1 That Is Functionally Redundant With Cln3–Cdk1

**FIGURE 4:**
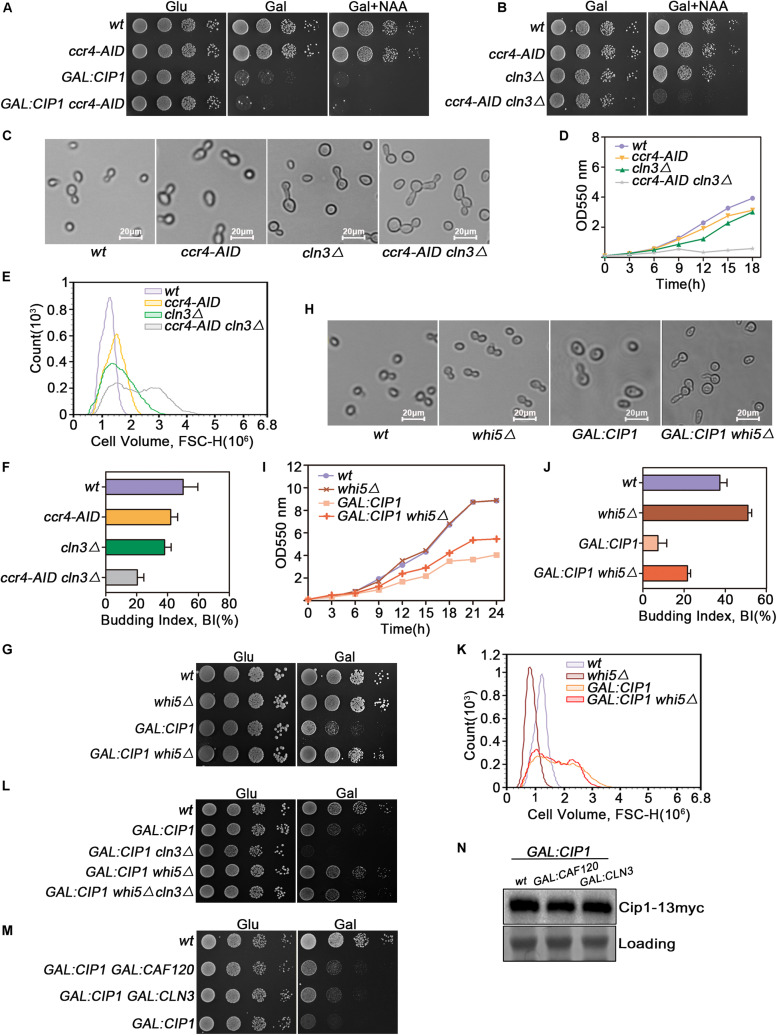
Cip1 acts as a dual suppressor of Cln3-Cdk1 and Ccr4-Caf120 to inhibit Whi5 function at START. **(A)** 10-fold serial dilutions from cultures of wild-type (YYJ138), *ccr4-AID* (YLP102), *GAL:CIP1* (YLP105), and *GAL:CIP1 ccr4-AID* (YLP106) cells were spotted onto YPD and YPG with or without 1.0 mM NAA. The auxin-inducible degron (AID) system was turned on by addition of auxin (NAA) in the presence of galactose. **(B)** 10-fold serial dilutions from cultures of wild-type (YYJ138), *ccr4-AID* (YLP102), *cln3*Δ (YLP107), and *ccr4-AID cln3*Δ (YLP108) cells were spotted onto YPG with or without 1.0 mM NAA. **(C)** The same strains were grown to mid-log phase in YPR. Galactose was added for 1 h, and samples were taken at the indicated time points after addition of 0.5 μM NAA. Cell morphology was visualized by microscopy (scale bar, 20 μm). **(D)** Growth curves of the indicated strains. **(E)** Flow cytometry analysis of the cell volume distributions for the indicated strains (20,000 cells analyzed/sample). **(F)** Budded cells were counted and expressed as a percentage of total cells. **(G)** Deletion of *WHI5* suppresses the cell-growth defect caused by Cip1 overexpression. 10-fold serial dilutions from cultures of wild-type (YFL3), *whi5*Δ (YLP23), *GAL:CIP1* (YFL97), and *GAL:CIP1 whi5*Δ (YLP75) cells were spotted onto YPD and YPG medium and incubated at 30°C for 3 days. **(H)** The same strains were grown to mid-log phase at 30°C and then shifted to YPG medium for 18 h. Samples were collected, and cell morphology was visualized by microscopy (scale bar, 20 μm). **(I)** Growth curves of the indicated strains. The indicated strains were grown in YPR. Galactose (2%) was added to induce overexpression of Cip1, and growth curves were generated. **(J)** Budded cells were counted and expressed as a percentage of total cells. **(K)** Flow cytometry analysis of the cell volume distributions for the indicated strains (20,000 cells analyzed/sample). **(L)** 10-fold serial dilutions from cultures of wild-type (YFL3), *GAL:CIP1* (YFL97), *GAL:CIP1 cln3*Δ (YXQ11), *GAL:CIP1 whi5*Δ (YLP75), and *GAL:CIP1 whi5*Δ *cln3*Δ (YLP78) cells were spotted onto YPD and YPG medium and incubated at 30°C for 3 days. **(M)** 10-fold serial dilutions from cultures of wild-type (YFL3), *GAL:CIP1* (YFL97), *GAL:CIP1 GAL:CLN3* (YLP174), and *GAL:CIP1 GAL:CAF120* (YLP175) cells were spotted onto YPD and YPG medium and incubated at 30°C for 3 days. **(N)** Western blot analysis of Cip1 protein expression under galactose induction in the indicated strains grown in YPG.

Because Ccr4 exhibited a robust interaction with Cip1, we investigated whether Ccr4 is a component of Cip1-mediated START control, redundant with Cln3. Auxin-induced Ccr4 depletion could not bypass growth inhibition induced by Cip1 overexpression (Figure 4A). Hence, we generated *ccr4-aid cln3*Δ double mutants and found that Ccr4 and Cln3 are synthetically lethal, consistent with the previously reported inviability of *ccr4*Δ *cln3*Δ double mutants (Figures 4B,C; Manukyan et al., 2008). Moreover, this synthetically lethality was resulted from G1/S transition defects, as the double mutant cells died with larger cell volume and defected budding compared to each single mutants (Figures 4C–F). Ccr4 is a non-essential gene but Auxin mediated depletion of Ccr4 protein displayed slow growth rate with abnormally large cells (Figures 4C–E).

### Deletion of *WHI5*, Which Is Downstream of Cln3 and Ccr4, Suppresses G1-Phase Arrest Caused by Overexpression of Cip1

Ccr4 negatively regulates the half-life of *WHI5* mRNA and hence influences the timing of G1/S cyclin transcription (Manukyan et al., 2008). Cln3-Cdk1, on the other hand, phosphorylates and inactivates Whi5 (Bruin et al., 2004). Hence, we asked whether deletion of *WHI5*, a common downstream target of both Ccr4 and Cln3, could restore growth to cells overexpressing Cip1. *GAL-CIP1 whi5*Δ cells had near-normal growth on plates (Figure 4G). Inhibition of budding and cell-cycle progression by Cip1 induction was significantly restored in the absence of Whi5 (Figures 4H–J). Intriguingly, cell buds elongated in *GAL:CIP1 whi5*Δ mutants (Figure 4H), which usually arises from a delayed switch from apical to isotropic bud growth, a process requiring M-CDK activity. Cip1 is an inhibitor of G1-CDK, however we could not rule out that Cip1 may exhibit inhibitory activity to Clb-Cdk1, specially in the absent of Whi5. This elongated cell buds in the mutants might be also explained by the defect of CLB5 and CLB6 expression when Whi5 is absent and G1/S transition is blocked by Cip1 overexpression.

The cell size distribution for *GAL:CIP1 whi5*Δ showed a shift to restored small cells compared to population of *GAL:CIP1* cells (Figure 4K). The relative mean value of *GAL:CIP1 whi5*Δ cell size is 1.68 × 10^6^, which is significantly smaller than 1.80 × 10^6^ of *GAL:CIP1* cells. We also generated *GAL:CIP1 whi5*Δ *cln3*Δ mutants and found that *Whi5* deletion could also restore the growth defect in the *GAL:CIP1 cln3*Δ background (Figure 4L).

Previous study reported that induction of *CLN3* in the Cip1 overexpressed cells partially restored the Cip1-dependent slow growth. To further support the idea that Ccr4-Caf120 works redundantly with Cln3-Cdk1, we generated *GAL:CIP1 GAL:CAF120* cells and compared the growth status with *GAL:CIP1 GAL:CLN3* cells. As shown in Figure 4M, simultaneous induction of *CAF120* could partially rescue the Cip1-dependent slow growth as well as induction of *CLN3.* This suppression is not because different overexpression extends of Cip1 in the compared strains, as the amount of Cip1 induced is identical as controlled in Figure 4N. Therefore, these data indicate that Ccr4 and Cln3, two crucial proteins upstream of Whi5, are both negatively regulated by Cip1.

**FIGURE 5:**
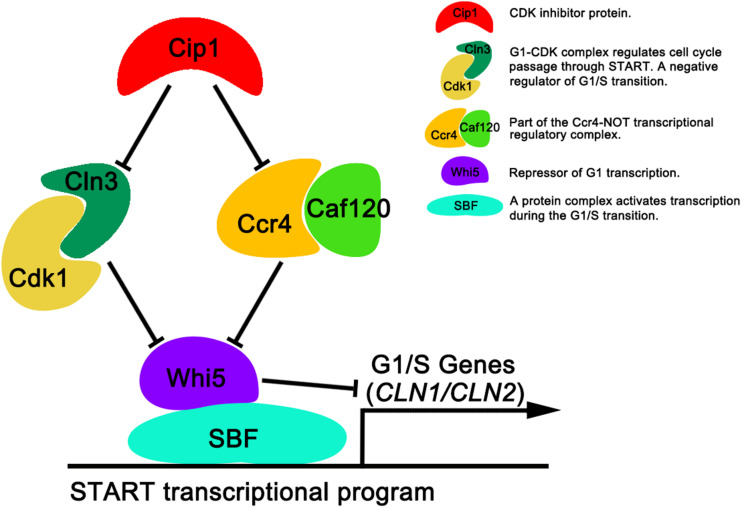
Dual repressive function of Cip1 on START timing via the redundant Cln3-Cdk1 and Ccr4-Caf120 pathways. The known interaction of Cip1 with the Cdk1-Cln3 complex inhibits its kinase activity. Ccr4 is a new target of Cip1, in parallel with Cln3-Cdk1. Dual inhibition by Cip1 down-regulates Whi5, preventing SBF from transcribing G1/S genes. The key description of each protein (protein complex) are shown.

In this study, we identified a new target of Cip1 that acts in parallel with Cln3 to regulate START. Our results are summarized in Figure 5. Two different pathways downstream of Cip1, mediated by Cln3-Cdk1 and Ccr4-Caf120, down-regulate *WHI5* through distinct mechanisms to inhibit the START transcriptional program. The previously reported interaction between Cip1 and the Cdk1-Cln3 complex inhibits G1-CDK kinase activity (Chang et al., 2017), whereas Ccr4 acts in parallel with Cln3-Cdk1 under the regulation of Cip1. Ccr4 regulates the size-dependent timing of *CLN2* mRNA expression by modulating the half-life of WHI5 mRNA. Importantly, a previous study of the regulation of START also reported that Ccr4 is redundant with Cln3-Cdk1 (Manukyan et al., 2008). Thus, our results help to explain why Cln3-Cdk1 is dispensable for cell-growth inhibition induced by Cip1 overexpression. Nevertheless, further study is required to explore the molecular details of Ccr4 inhibition by Cip1.

In summary, our observations reveal that Cip1 acts as a dual repressor to negatively regulate both Cln3-Cdk1 and Ccr4 complex, with the final result of maintaining Whi5 active, thereby preventing SBF from transcribing G1/S genes. The situation in yeast may be conserved in other organisms, including humans. Notably in this regard, the Ccr4-Not complex in human cells was recently implicated in cancer development (Allison, 2017; Maolake et al., 2017; Vicente et al., 2018). Accordingly, it would be worthwhile to determine whether p21 is involved in a similar control mechanism in human cells.

## Experimental Procedures

### Constructs, Strains, Culture Conditions and Cell Size Analysis

All strains used in this work were derived from *Saccharomyces cerevisiae* W303-1a (Thomas and Rothstein, 1989) and listed in Table 1. Strains were generated following standard procedures by integration of PCR-generated cassettes as previously described (Zeng et al., 2018). Plasmids constructed for this study were validated by sequencing and are listed in Table 2. Cells were grown in standard media containing 1% yeast extract, 2% peptone, and 2% glucose. For induction experiments, cells were grown overnight in media containing 2% raffinose, and then inoculated into 2% galactose media or onto galactose plates with or without 1-naphthylacetic acid (NAA) as indicated. Fluorescent Activated Cell Sorting (FACS) was used in this study for the analysis of the cell size distribution in a given cell population. 20,000 cells were read for each profile on a NovoCyte D3130 (ACEA) instrument. The number of cells (y-axis) vs. values of the forward scatter signal band FSC-H (*x*-axis) was represented for cell size analysis.

**TABLE 1:** Yeast strains in this study.

Strain	Genotype	Source
W303-1a	*MATa ade2-1 ura3-1 his3-11*,*15 trp1-1 leu2-3*,*112 can1-100*	Thomas and Rothstein, 1989
YFL3	W303-1a *bar1*Δ	Ren et al., 2016
YXQ7	W303-1a *bar1*Δ *cln3△*:*TRP1*	This study
YFL97	W303-1a *bar1*Δ *GAL-CIP1-13myc*:*URA3*	Ren et al., 2016
YXQ11	W303-1a *bar1*Δ *GAL-CIP1-13myc*:*URA3 cln3*Δ:*TRP1*	This study
YZZ6	W303-1a *bar1*Δ *CLN2-3HA*:*KanMX6*	This study
YZZ20	W303-1a *bar1*Δ *cip1*Δ:*HIS3 CLN2-3HA*:*KanMX6*	This study
YXQ8	W303-1a *bar1*Δ *cln3*Δ:*TRP1 CLN2-3HA*:*KanMX6*	This study
YXQ9	W303-1a *bar1*Δ *cip1*Δ:*HIS3 cln3*Δ:*TRP1 CLN2-3HA*:*KanMX6*	This study
YLP139	W303-1a *bar1*Δ *CLN2-3HA*:*KanMX6 SIC1-13myc*:*URA3*	This study
YLP149	W303-1a *bar1*Δ *cip1*Δ:*HIS CLN2-3HA*:*KanMX6 SIC1-13myc*:*URA3*	This study
YLP155	W303-1a *bar1*Δ *cln3*:*TRP1 CLN2-3HA*:*KanMX6 SIC1-13myc*:*URA3*	This study
YLP138	W303-1a *bar1*Δ *cip1*Δ:*HIS3 cln3*Δ:*TRP1 CLN2-3HA*:*KanMX6 SIC1-13myc*:*URA3*	This study
YFL100	W303-1a *bar1*Δ *CIP1-13myc*:*URA3*	Ren et al., 2016
YLP41	W303-1a *bar1*Δ *CIP1-13myc*:*URA3 CCR4-3HA*:*KanMX6*	This study
YLP42	W303-1a *bar1*Δ *CIP1-13myc*:*URA3 CAF120-3HA*:*KanMX6*	This study
YYJ138	W303-1a *bar1*Δ *GAL-OsTIR1*:*URA3*	This study
YLP102	W303-1a *bar1*Δ *GAL-OsTIR1*:*URA3 CCR4-AID-9myc*:*HPH*	This study
YLP105	W303-1a *bar1*Δ *GAL-OsTIR1*:*URA3 GAL-CIP1-13myc*:*HIS3*	This study
YLP106	W303-1a *bar1*Δ *GAL-OsTIR1*:*URA3 CCR4-AID-9myc*:*HPH GAL-CIP1-13myc*:*HIS3*	This study
YLP107	W303-1a *bar1*Δ *GAL-OsTIR1*:*URA3 cln3*Δ:*TRP1*	This study
YLP108	W303-1a *bar1*Δ *GAL-OsTIR1:URA3 CCR4-AID-9myc:HPH cln3*Δ*:TRP1*	This study
YLP23	W303-1a *bar1*Δ *whi5*Δ:*URA3*	This study
YLP75	W303-1a *bar1*Δ *GAL-CIP1-13myc*:*URA3 whi5*Δ:*HIS3*	This study
YLP78	W303-1a *bar1*Δ *GAL-CIP1-13myc:URA3 whi5*Δ:*HIS3 cln3*Δ:*TRP1*	This study
YLP174	W303-1a *bar1*Δ *GAL-CLN3-HA*:*HIS3 GAL-CIP1-13myc*:*URA3*	This study
YLP175	W303-1a *bar1*Δ *GAL-CAF120-HA*:*HIS3 GAL-CIP1-13myc*:*URA3*	This study

**TABLE 2:** Plasmids used in this study.

Plasmid	Base plasmid/Genotype	Source
pGADT7	amp^r^/LEU2	Lab stock
pGBKT7	kan^r^/TRP1	Lab stock
PLP56	amp^r^/URA3 pRS306-GAL-13myc	Lab stock
PLP110	amp^r^/HIS3 pRS303	Lab stock
PLP111	amp^r^/TRP1 pRS304	Lab stock
PLP113	amp^r^/URA3 pRS306	Lab stock
PLP130	amp^r^/URA3 pRS306-GAL-CIP1-13myc	Lab stock
PLP132	kan^r^/amp^*r*^/G418 pFA6a-3HA-KanMX6	Lab stock
PLP139	amp^r^/LEU2 GAL4-AD-CIP1	This study
PLP140	kan^r^/TRP1 GAL4-BD-CIP1	This study
PLP142	kan^r^/TRP1 GAL4-BD-CCR4	This study
PLP149	amp^r^/LEU2 GAL4-AD-CAF120	This study
PLP183	amp^r^/LEU2 GAL4-AD-CAF40	This study
PLP184	amp^r^/LEU2 GAL4-AD-CDC36	This study
PLP185	amp^r^/LEU2 GAL4-AD-NOT5	This study
PLP186	amp^r^/LEU2 GAL4-AD-MOT2	This study
PLP187	amp^r^/HPH pHYD-AID-9myc	Lab stock
PLP188	amp^r^/HIS3 pRS303-GAL-CIP1-13myc	Lab stock
PLP191	amp^r^/LEU2 GAL4-AD-NOT3	This study
PLP196	kan^r^/TRP1 GAL4-BD-hP21	This study
PLP201	amp^r^/LEU2 GAL4-AD-hCCR4	This study

### Co-IP and Western Blotting

For co-immunoprecipitation (Co-IP) and immunoprecipitation, 10^9^ cells were collected and extracted by glass bead beating in lysis buffer [50 mM Tris–HCl, pH 7.8, 150 mM NaCl, 1 mM EDTA, 10% (v/v) glycerol, and 1% (v/v) Triton X-100] supplemented with protease inhibitors (1 mM PMSF, 0.15 mM aprotinin, 1 mM leupeptin, and 1 mM pepstatin) and phosphatase inhibitors (0.5 mM sodium pyrophosphate, 2 mM NaF, and 2 mM β-glycerophosphate). Twenty microliters of anti-HA HA-7 (Sigma A7470, mouse monoclonal purified IgG) or anti-myc HA-7 agarose matrix (Sigma A2095, mouse monoclonal purified IgG) was added to extracts to immunopurify proteins of interest C-terminally tagged with 13myc or 3HA, respectively. The samples were gently rotated for 1 h at 4°C, after which the beads were recovered by centrifugation at 500 × *g* for 1 min at 4°C, and the supernatants were discarded. The beads were then washed three times with 1 mL cold lysis buffer. Finally, the proteins were released by boiling the beads in Laemmli sample buffer. Enriched proteins were resolved by SDS-PAGE and subjected to western blotting.

Whole-cell extracts for western blotting were prepared by glass bead beating in trichloroacetic acid (TCA) and then resolved by SDS-PAGE as previously described. The primary antibodies used in this study were anti-HA (Sigma A7470, mouse monoclonal purified IgG) and anti-myc (Sigma A2095, mouse monoclonal purified IgG).

### Large Scale Co-IP for Mass Spectroscopy

To identify the interacting proteins of Cip1 by mass spectroscopy proteomic analysis, a larger scale of co-immunoprecipitation procedure was followed. Around 10 g of cells expressing Cip1-13myc from its own promoter were collected and extracted. Upon extraction, samples were clarified for 1 h at 100,000 × *g* in a Thermo ultracentrifuge (Sorvall COMB PLUS). Extracts were then incubated with 100 μl of pre-equilibrated anti-myc HA-7 agarose matrix (Sigma A2095, mouse monoclonal purified IgG), and rotated gently for 1 h at 4°C. After that, beads were washed 6 times with lysis buffer and the proteins from the immunoprecipitate were released from the beads by mixing with 50 μl of room temperature 8M urea in 100 mM Tris pH 8.0 for 5 min. After releasing, the copurified proteins from the sample were separated by SDS-PAGE and stained with Coomassie Brilliant blue, followed by detection by mass spectrometry. As a control, a mock IP was performed using cells without myc-tagged Cip1 protein.

### Yeast Two-Hybrid Assay

To investigate the interactions of Cip1 with yeast Ccr4–Not and p21 with human Ccr4, the Gal4-based Matchmaker Yeast Two-hybrid System 3 (Clontech Laboratories, Mountain View, CA, United States) was used. The protocol was essentially as previously described (Zeng et al., 2018). Briefly, the indicated genes, amplified from a budding yeast or a human cDNA library, were fused to the GAL4 activation domain (AD) in vector pGADT7 or the GAL4 DNA-binding domain (BD) in pGBKT7. Pairs of corresponding constructs were co-transformed into the tester strain AH109, in which the HIS3 and ADE2 reporter genes are under the control of three completely heterologous GAL4-responsive UAS, and promoter elements GAL1 and GAL2, respectively. Transformants were first grown on SD/-Leu/-Trp medium and subsequently plated on SD/-Ade/-His/-Leu/-Trp medium.

### RNA Extraction and qRT-PCR

Around 10^9^ cells were harvested for each sample and washed once with pre-chilled diethyl pyrocarbonate (DEPC)-treated water. Cell pellets were snap-frozen in liquid nitrogen and stored at −80°C prior to RNA extraction. Total RNA was obtained using the Trizol reagent (Invitrogen). RNA was then reverse transcribed using the PrimeScript RT kit (Takara, RR014A). The cDNA library was used as a template in real-time PCR using the Takara SYBR Premix Ex-Taq (Tli RNase H Plus) kit (Takara, RR420A). The primers for *CLN2* and *ACT1* were the same as in our previous study (Zhang Z. et al., 2017).

### Statistics

All values were expressed as the mean ± SD, and statistical analyses were carried out using Student’s *t*-test.

## Data Availability Statement

The datasets generated for this study are available on request to the corresponding author.

## Author Contributions

FZ, PL, XL, and ZH conceived and designed the experiments. PL, XL, ZH, and XZ performed the experiments. FZ, PL, ZH, and JD analyzed the data. FZ and PL wrote the manuscript. All authors contributed to the article and approved the submitted version.

## Conflict of Interest

The authors declare that the research was conducted in the absence of any commercial or financial relationships that could be construed as a potential conflict of interest.
